# Colloidal Synthesis of Multinary Alkali-Metal Chalcogenides Containing Bi and Sb: An Emerging Class of I–V–VI_2_ Nanocrystals with Tunable Composition and Interesting Properties

**DOI:** 10.1021/acs.chemmater.3c00673

**Published:** 2023-06-08

**Authors:** Nilotpal Kapuria, Bingfei Nan, Temilade Esther Adegoke, Ursel Bangert, Andreu Cabot, Shalini Singh, Kevin M. Ryan

**Affiliations:** † Department of Chemical Sciences and Bernal Institute, University of Limerick, V94T9PX Limerick, Ireland; ‡ Catalonia Institute for Energy Research -IREC, 08930 Barcelona, Spain; § ICREA, 08010 Barcelona, Spain; ∥ Department of Physics and Energy and Bernal Institute, University of Limerick, V94T9PX Limerick, Ireland

## Abstract

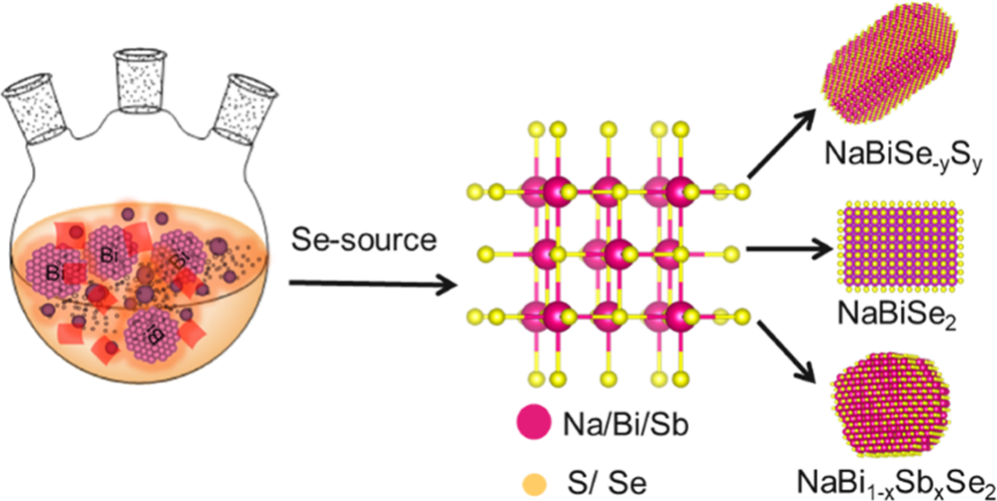

The growth mechanism and synthetic controls for colloidal multinary metal chalcogenide nanocrystals (NCs) involving alkali metals and the pnictogen metals Sb and Bi are unknown. Sb and Bi are prone to form metallic nanocrystals that stay as impurities in the final product. Herein, we synthesize colloidal NaBi_1–*x*_Sb*_x_*Se_2–*y*_S*_y_* NCs using amine–thiol–Se chemistry. We find that ternary NaBiSe_2_ NCs initiate with Bi^0^ nuclei and an amorphous intermediate nanoparticle formation that gradually transforms into NaBiSe_2_ upon Se addition. Furthermore, we extend our methods to substitute Sb in place of Bi and S in place of Se. Our findings show the initial quasi-cubic morphology transforms into a spherical shape upon increased Sb substitution, and the S incorporation promotes elongation along the <111> direction. We further investigate the thermoelectric transport properties of the Sb-substituted material displaying very low thermal conductivity and n-type transport behavior. Notably, the NaBi_0.75_Sb_0.25_Se_2_ material exhibits an ultralow thermal conductivity of 0.25 W·m^–1^·K^–1^ at 596 K with an average thermal conductivity of 0.35 W·m^–1^·K^–1^ between 358 and 596 K and a *ZT*_max_ of 0.24.

## Introduction

Several multinary metal chalcogenides have been identified as high-performance materials in diverse fields such as photovoltaics, thermoelectrics, optoelectronics, and catalysis.^1−9^ Among them, the coinage metals (Cu, Ag)-based I–V-VI_2_ NCs (V = As, Sb, As) have recently gained attention owing to their promising chemical and physical properties to be used as thermoelectric (TE) materials and as absorber layers in solar cells among other applications.^10−12^ Replacing the Valence I coinage metals with main group alkali metals such as Na and K and retention of the V valence pnictogen metals give rise to compositions with potentially useful properties such as high light absorption coefficients and intrinsically low thermal conductivities.^13−17^ The nonparticipation of low energy orbitals of alkali metals in the valence band is further beneficial to tune TE and photovoltaic properties.^15^ The presence of group V metals (Sb, Bi) in a trivalent state allows local structural anharmonicity induced by stereochemically active lone pairs (LPs) that is crucial to achieving low thermal conductivity.^18^ Besides, the substitution of foreign cations such as Sb on the Bi sites increases the configuration entropy and point defects with efficient phonon scattering to lower the thermal conductivity value.^19^ However, very high configurational entropy is detrimental to charge carrier transport, reducing electrical conductivity. Thus, the Sb to Bi substitution concentration needs to be optimized in such main group I–V–VI_2_ compositions to simultaneously reach low thermal and high electrical conductivities.

In the colloidal hot injection (HI) method, efficient control over nucleation and growth stages can be achieved by choosing suitable precursors and reaction parameters.^20−22^ Several multinary metal chalcogenide compositions such as Cu_2_FeSnSe_4_, Cu_2_MSnS_4_ (M = Co, Fe, Ni, Zn, Cd), Cu_2_ZnSn(S_1–*x*_Se*_x_*)_4_, CuIn_1–*x*_Gax(S_1–*y*_Se*_y_*)_2_, Cu_α_Zn_β_Sn_γ_Se_δ_, Ag-In-Zn-S, etc. have been achieved in NC forms by using the HI method.^4,23−28^ The compositional library has been further extended using attractive cation exchange (CE) processes.^29−31^ For the above-mentioned systems, Ag_2_E or Cu_2_E (E = S, Se, Te) form as initial nuclei possessing highly mobile Ag^+^ or Cu^+^ cations on the rigid chalcogen sublattice. The subsequent cationic substitution into the ion-conducting sublattice forms multinary phases.^32−35^ Systems involving trivalent group VA metals tend to form monometallic NCs (e.g., Bi^0^) as the initial nuclei in the presence of reducing solvents such as alkyl amine. The gradual transformation of these monometallic NCs to compound metal chalcogenide compositions may be possible at elevated temperatures and in the presence of mobile Ag^+^ or Cu^+^.^21^ However, this type of transformation in the alkali metal-based chalcogenide system is yet to be thoroughly investigated. Very recently, using highly reactive and flammable metal hydride precursors, phase-pure ternary NaBiSe_2_ and NaSbSe_2_ NCs were synthesized.^36,37^ The high reactivity of the metal hydrides leads to uncontrolled nucleation and growth kinetics limiting control over size, and thus, wide size distributions were obtained. The complexity further increases with compositions consisting of three or more elements with the propensity of co-formation of binary chalcogenide phases. Overall, tuning the shape and composition with uniformity in multinary alkali metal chalcogen systems is an extremely challenging task.

Herein, we develop a colloidal HI approach using a thiol–amine solution of Se as a precursor to synthesize multinary alkali metal-based NCs containing Sb and Bi. The easily processed thiol–amine solution of selenium is highly reactive to dissolve metallic Bi NCs. The versatility of the approach allowed the synthesis of NaBi_1–*x*_Sb*_x_*Se_2_ NCs with complete composition and shape tunability. Finally, we studied the TE performance of the Sb-substituted materials. The best TE performance was obtained from NaBi_0.75_Sb_0.25_Se_2_ NCs which exhibited ultralow thermal conductivity and a promising TE figure of merit.

## Results and Discussion

NaBiSe_2–*y*_S*_y_* NCs were produced using a HI colloidal synthesis approach (see the detailed procedure in the Experimental Section). Briefly, in a typical reaction, two equivalents of sodium oleate (Na–OL) solution were mixed with an equivalent of pnictogen metal (Bi, Sb) acetate salt in oleylamine (OLA) and 1-octadecene (ODE) solvent mixture and evacuated at 105 °C for 1h. Subsequently, 3 mL of 0.5 M Se-alkahest stock solution was injected at 200 °C under the Ar atmosphere as the chalcogen source.

**Figure 1:**
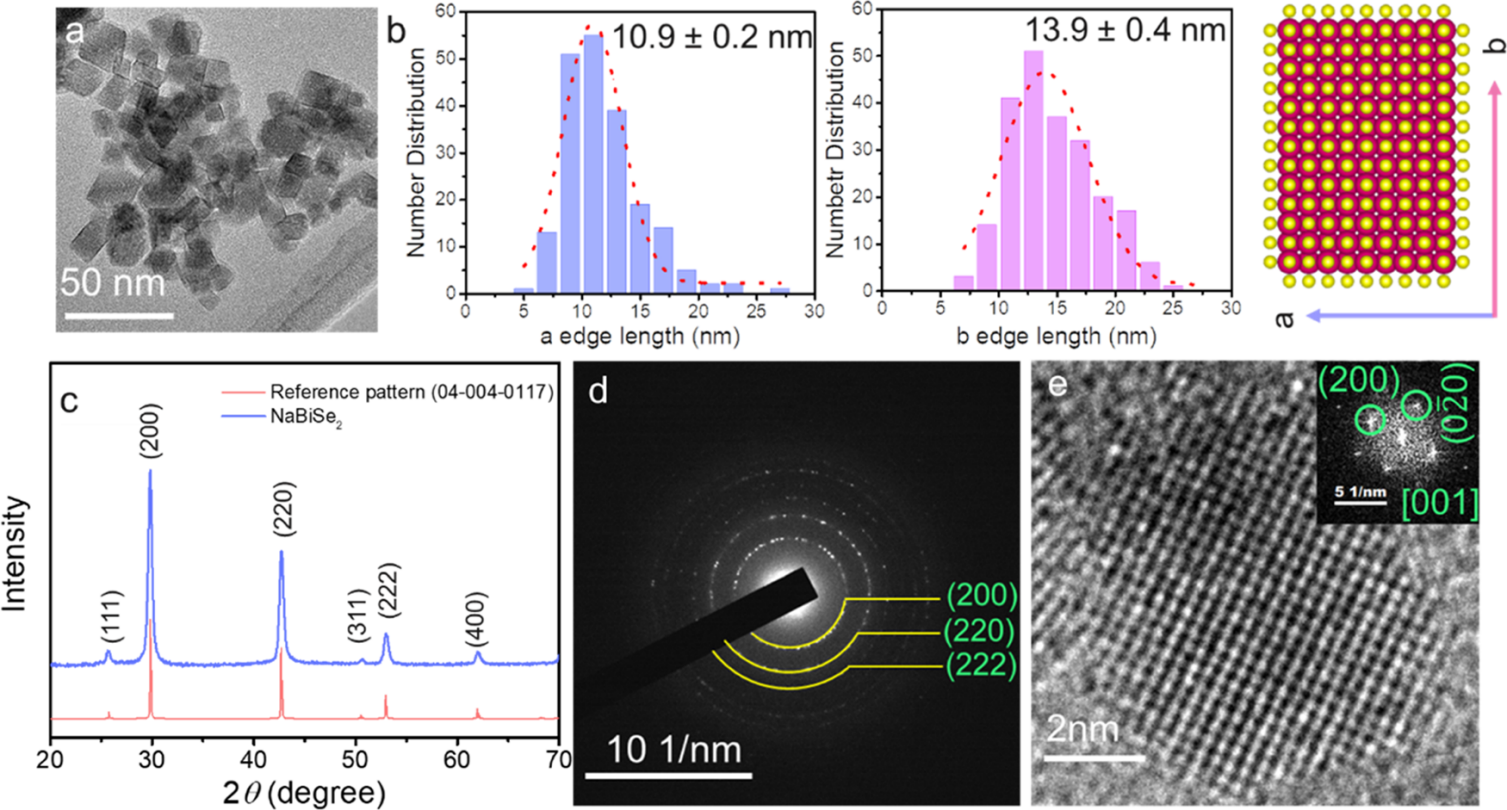
(a) Low magnification transmission electron microscopy (TEM) image of (b) size distribution, (c) X-ray diffraction (XRD) pattern, (d) selected area electron diffraction (SAED) pattern, NaBiSe_2_ NCs, and (e) high-resolution TEM of a NaBiSe_2_ NC and the inset shows fast Fourier transform (FFT) of the corresponding HRTEM.

Low magnification transmission electron microscopy (TEM) images show the as-synthesized NaBiSe_2_ NCs to be quasi-cubic in shape with an average size of below ∼14 nm (Figures 1a,b and S1a,b). The powder X-ray diffraction (PXRD) analysis of the ternary NCs (Figure 1c) shows them to adopt the rock salt crystal (*Fm*-3*m*) structure with an average crystallite size of ∼12 nm as calculated from Rietveld refinement (Figure S1). The average crystallite size is close to the size obtained from TEM observations, implying the particles are single crystals. The high crystallinity is further confirmed by the analysis of the selected area electron diffraction (SAED) pattern (Figure 1d) and fast Fourier transform of a high-resolution TEM (HRTEM) image displaying a d-spacing value of ∼3.0 Å for the (200) planes of the rock salt NaBiSe_2_ NCs (Figure 1e).

**Figure 2:**
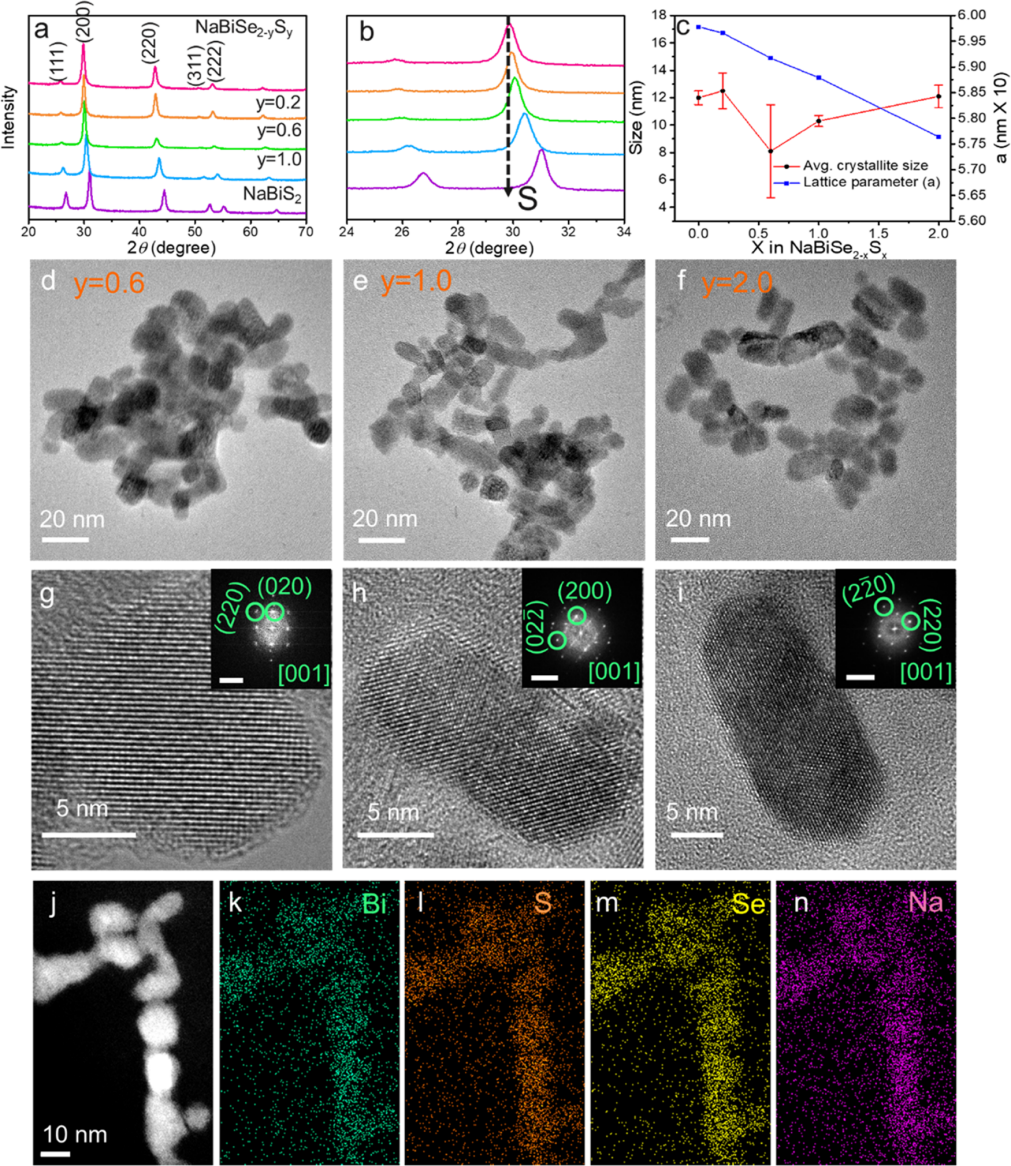
(a) XRD patterns of NaBi*_x_*Se_2–*y*_S*_y_* NCs and (b) 2*θ* shifts of (200) and (111) peaks. (c) S substitution-dependent average crystallite size and lattice parameter obtained from Rietveld refinement of the XRD pattern. TEM images of (d) NaBiSe_1.4_S_0.6_, (e) NaBiSe_1_S_1_, and (f) NaBiS_2_. HRTEM of (g) NaBiSe_1.4_S_0.6_, (h) NaBiSe_1_S_1_, and (i) NaBiS_2_. STEM-EDS elemental maps for (j) NaBiSe_1_S_1_ NCs showing (k) Bi, (l) S, (m) Se, and (n) Na.

For S substitution, an excess concentration of elemental S was mixed with Se thiol–amine solution to formulate the chalcogen source (detailed in the Experimental Section). The increased substitution of S induces anisotropy in shape with elongation along the <111> direction. This elongation is confirmed by the increased intensity of the XRD peaks associated with the {111} sets of planes (Figure 2a). The cation and anion sublattices are stacked alternately along the <111> direction.^16^ Thus, the substitution of S occurs along the <111> direction resulting in elongation. As per Vegard’s law, increased S substitution displays an XRD peak shift toward higher 2*θ* values (Figure 2a,b). The lattice parameter calculated from Rietveld refinement (Figure S2) also agrees with the above observation, where 50% S substitution reduced the lattice parameter by ∼16% compared to a Se-rich phase (Figure 2c). Low magnification TEM images display the anisotropic shape change where NaBiS_2_ NCs are rod-shaped (Figure 2d–f). HRTEM analysis (Figure 2g–i) further confirms the gradual reduction in the lattice parameter where the d-spacing is ∼3.0 Å for {002} sets of planes of Se-rich NCs compared to ∼2.9 Å for {002} sets of planes of S-rich NCs. Furthermore, STEM-EDS elemental maps of the 50% S-substituted NCs displayed the presence of Na, Bi, S, and Se distributed homogenously in the NCs (Figure 2j–n).

**Figure 3:**
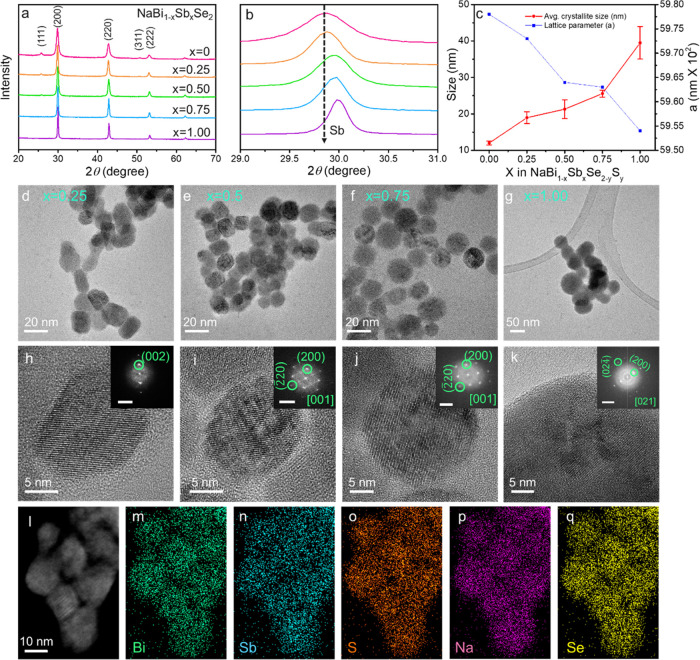
(a) XRD patterns of NaBi_1-x_Sb_x_Se_2_ NCs and (b) 2*θ* shift of the (200) peak. (c) Sb substitution-dependent average crystallite size and lattice parameter obtained from Rietveld refinement of the XRD pattern. TEM images of (d) NaBi_0.75_Sb_0.25_Se_2_, (e) NaBi_0.50_Sb_0.50_Se_2_, (f) NaBi_0.25_Sb_0.75_Se_2_, and (g) NaSbSe_2_. HRTEM of (h) NaBi_0.75_Sb_0.25_Se_2_, (i) NaBi_0.50_Sb_0.50_Se_2_, (j) NaBi_0.25_Sb_0.75_Se_2_, and (k) NaSbSe_2_. STEM-EDS elemental maps for (l) NaBi_0.50_Sb_0.50_Se_2_ NCs showing (m) Bi, (n) Sb, (o) S, (p) Na, and (q) Se.

The versatility of the synthesis approach allows the extension of the NCs composition to multinary NaBi_1–*x*_Sb*_x_*Se_2_ by Sb substitution in Bi sites. A series of NaBi_1-x_Sb_x_Se_2_ NCs with varied stoichiometry were synthesized by increasing the growth temperature to 240 from 200 °C (Se addition at 200 °C) to aid Bi^3+^ replacement with Sb^3+^ and increasing the growth time >36 minutes depending on the Sb to Bi percentage, as detailed in the Experimental Section. The elemental composition is confirmed by ICP-OES analysis (Table S1). Bi:Sb composition ratios follow the metal precursor mole ratios used in the reaction. With increased Sb substitution, the XRD peaks shift to higher 2*θ* values, accompanied by a narrowing of peak width as the stoichiometry changes from Bi rich to Sb rich, supporting the Sb incorporation (Figure 3a,b). The lattice constant (calculated from Rietveld refinement of NaBi_1–*x*_Sb*_x_*Se_2_ NCs, Figure S3) reduces with increased Sb substitution in agreement with Vegard’s law. The Sb substitution increases the crystallite size from ∼12 nm for NaBiSe_2_ to ∼40 nm for NaSbSe_2_ NCs (Figure 3c). The shape anisotropy of the multinary NCs gets affected by Sb substitution forming spherical NCs for Sb-rich phases (Figure 3d-g). Sb^3+^ substitution in place of Bi^3+^ will require Bi^3+^ expulsion from the lattice. The increased growth temperature increases the cationic diffusion, which triggers the replacement and rearrangement of the crystal structure to form quasi-spherical shapes, similar to the thermodynamically favored cuboctahedral shape for FCC crystals.^38^ The HRTEM of these NCs confirms their cubic crystal structure and depicts a clear visual of the shape change (Figure 3h,i). Furthermore, the homogenous distribution of Na, Sb, Bi, S, and Se is confirmed via STEM-EDS elemental mapping of NaBi_0.5_Sb_0.5_Se_2_ NCs (Figure 3l–q).

**Figure 4:**
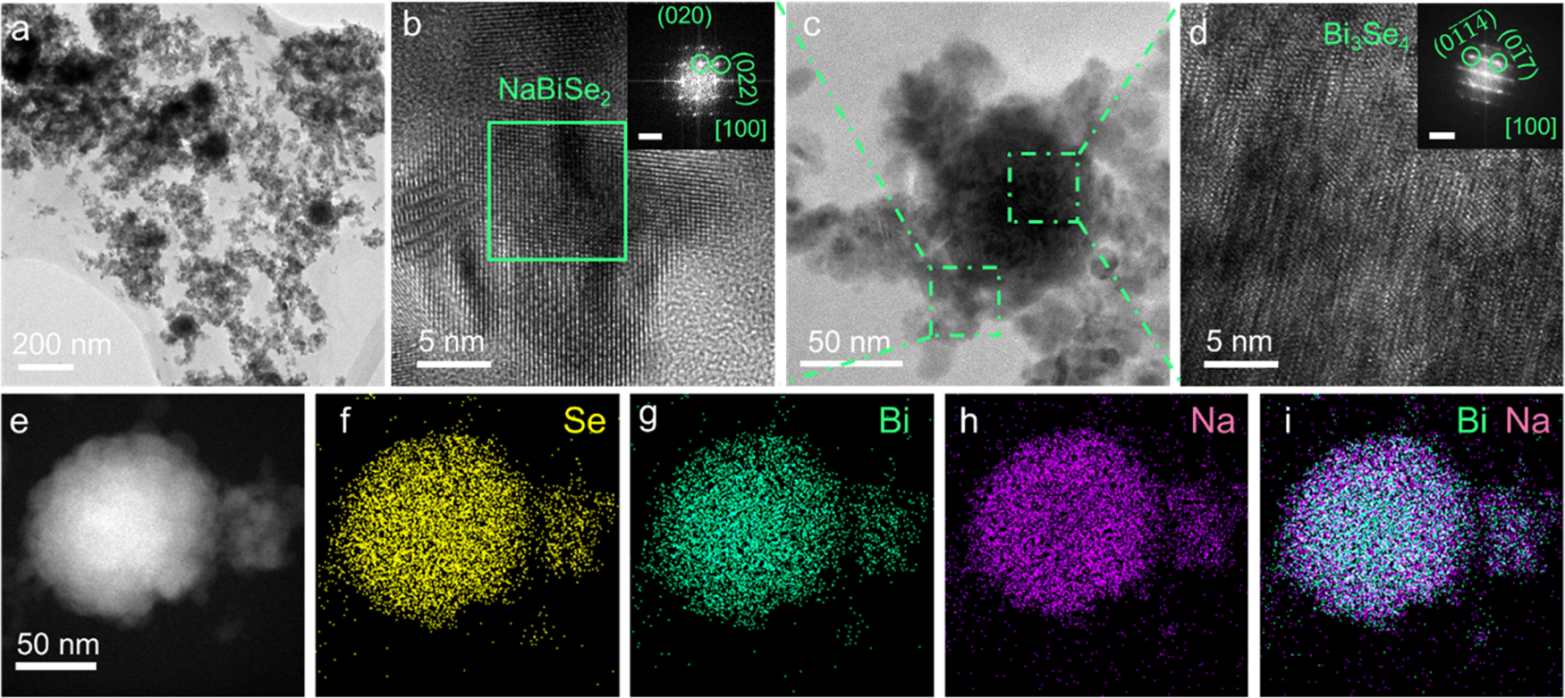
(a) Low magnification TEM image of the aliquot collected after 30 seconds of Se introduction. (b) HRTEM image of the NaBiSe_2_ NC present on the peripheral segment of the (c) larger NCs and HRTEM of (d) the core of the larger NCs. The insets show the FFT pattern of the corresponding HRTEM with a scale bar of 5 1/nm. (e) STEM-EDS elemental maps of the larger NCs accompanied with elemental maps for (f) Se, (g) Bi, (h) Na, and (i) overlay of Na and Bi.

OLA is known to reduce the pnictogen–metal salts into metallic NCs, as we have previously reported in the formation of Bi^0^ using BiCl_3_ precursor with OLA.^21,22^ Here, the Bi NCs form upon the reduction of bismuth acetate by OLA. The presence of aldimine (which forms upon oxidation of OLA) in the aliquot supernatant collected at 200 °C before Se addition from the NaBiSe_2_ reaction, showing peaks at ∼3.2 and ∼7.6 ppm in the ^1^H NMR (Figure S4), confirms the OLA induced reduction of Bi(OAc)_3_. TEM analysis of the aliquot withdrawn before Se introduction displays the presence of quasi-cubic nanoparticles alongside crystalline Bi^0^ NCs (Figure S5). The quasi-cubic nanoparticles are unstable under the electron beam and display amorphous features in the selected area electron diffraction pattern (Figure S6). The lower stability of these disordered nanoparticles under ambient conditions renders further characterization difficult. The formation of NaBiSe_2_ NCs starts immediately after Se-stock injection, which is confirmed from the XRD (Figure S8) analysis of the aliquot withdrawn after 30 s of Se introduction, showing characteristic peaks from Bi (Rhombohedral, R3̅m), rock salt NaBiSe_2_ NCs and Bi_3_Se_4_ coinciding with the main peaks of Bi and NaBiSe_2_. TEM images (Figure 4a) of the aliquot sample withdrawn 30 seconds after Se addition display the presence of tiny NCs alongside larger secondary NCs. It can also be observed that the tiny NCs are aggregated on the periphery of the larger secondary NCs. HRTEM characterization of the NCs present (Figure 4b) on the peripheral segment of a larger NC (Figure 4c) exhibits d-spacing values of 3.0 and 2.1 Å matching well with (020) and (022) planes of rock salt NaBiSe_2_. From the same aliquot sample, HRTEM analysis of a detached NC shows phase conformity with rock salt NaBiSe_2_ crystals (Figure S9). HRTEM analysis of the large secondary NCs confirms that they are Bi_3_Se_4_ (*R*-3*m*) with d-spacing values of 3.1 and 2.3 Å for (01̅7) and (01 14) planes, respectively (Figures 4b and S10). STEM-EDS elemental mapping (Figure 4e–i) of a large NC displays complete selenization (Figure 4f) with the Na-deficient core with a Na-rich periphery (Figure 4i), indicating the nascent NaBiSe_2_ NCs are aggregated at the periphery of Bi_3_Se_4_.

**Scheme 1:**
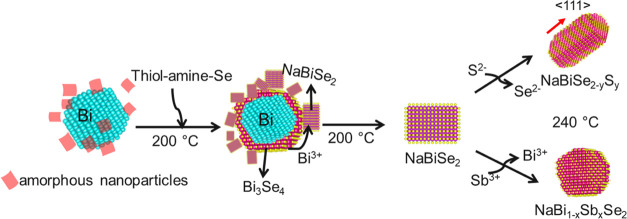
Illustration of NaBiSe_2_, NaBiSe_2–*y*_S*_y_*, and NaBi_1–*x*_Sb*_x_*Se_2_ NC Formation Starting from Bi NCs

The XRD patterns of the aliquots withdrawn at different growth times between 30 seconds to 20 minutes (Figure S8) show the formation of NaBiSe_2_ NCs alongside decreasing intensity of the Bi phase, which ultimately diminishes after 20 min of growth time at 200 °C. However, the discernible intensity of the Bi_3_Se_4_ phase can only be seen in the 30 seconds aliquots, confirming the rapid transformation after Se addition. Thus, we propose that the amorphous quasi-cubic-shaped nanoparticles are initially formed alongside Bi NCs (Scheme 1). These amorphous NPs will possibly (amorphous NPs contain Na as suggested by the presence of a peak at ∼1071 eV for Na^+^ in the XPS survey of 200 °C aliquot in Figure S15) act as the intermediates for further transformation. The introduction of Se converts the Bi NCs into Bi_3_Se_4_ and acts as reservoirs of Bi^3+^. In nonclassical growth theory, a transition between transient amorphous or disordered phase to crystalline phase is observed.^39,40^ Atomic rearrangement during cationic diffusion can lead to crystalline phase formation from the amorphous phase. Thus, the diffusion of Bi^3+^ from the Bi_3_Se_4_ phase into the amorphous (disordered) intermediate converts it into NaBiSe_2_ NCs. Increased growth time at 200 °C allows more Bi^3+^ diffusion to form phase-pure NaBiSe_2_ NCs. Consequently, the larger Bi and Bi_3_Se_4_ NCs dissolve as the growth progresses. Similarly, for the NaBi_0.5_Sb_0.5_Se_2_ NCs, the TEM analysis of the aliquot withdrawn at 200 °C before Se addition exhibits an amorphous phase alongside Bi and Sb NC formation (Figure S11). The XRD pattern of the aliquot withdrawn 30 seconds after Se addition displays the formation of NaBiSe_2_ NCs alongside the presence of unconverted Sb NCs (Figure S12a). When the aliquot is observed under TEM, the Bi_3_Se_4_ phase formation is revealed, as seen for NaBiSe_2_ NCs (Figure S12b,d). The XRD pattern from the aliquot collected 5 min after Se addition at 220 °C indicates the complete conversion of the Sb NCs (Figure S12a). Thus, the transformation of Sb NCs, possibly into an Sb-chalcogenide phase, acts as the source of Sb^3+^ diffusing into NaBiSe_2_ at a higher temperature of 240 °C to form NaBi_1-x_Sb_x_Se_2_ NCs.

**Figure 5:**
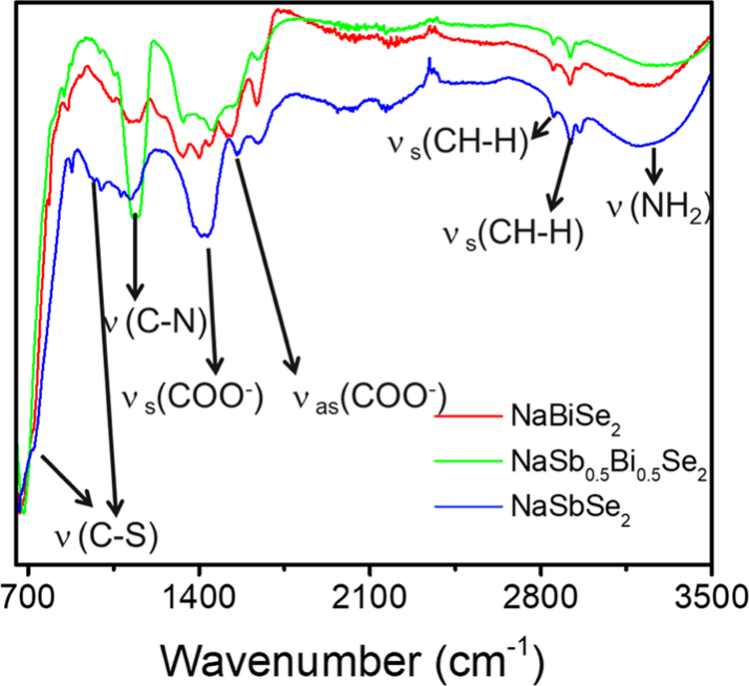
IR spectra of NaBiSe_2_ (red), NaSb_0.5_Bi_0.5_Se_2_ (green), and NaSbSe_2_ (blue) nanocrystal powder.

To understand the surface chemistry of the solid solution nanocrystals, IR spectra of the NaBiSe_2_, NaBi_0.5_Sb_0.5_Se_2_, and NaSbSe_2_ NC powders are analyzed (Figure 5). In the IR spectra for all the NCs, two strong bands at ∼1440 (symmetric) and ∼1550 cm^–1^ (asymmetric) are displayed, which are characteristic vibrational features from the surface-bound carboxylates (COO^–^) from the oleate species.^41^ The difference between the symmetric and asymmetric bands is around ∼110 cm^–1^, which signifies the bidentate nature of the surface-bound oleate.^36^ The surface-bound oleylamine exhibits its characteristic peaks at ∼1150 cm^–1^ (C–N stretching), 1650 cm^–1^ (N–H bending), and a broad stretching band around ∼3250 cm^–1^ coming from NH_2_ stretching.^42,43^ Additionally, the C–S stretching frequencies between 1050 and 650 cm^–1^ could arise from a surface-bound alkane thiol–selenium complex formed upon the reaction of thiol–amine solution with Se, as per equation 2 mentioned in Figure S16.^44,45^

**Figure 6:**
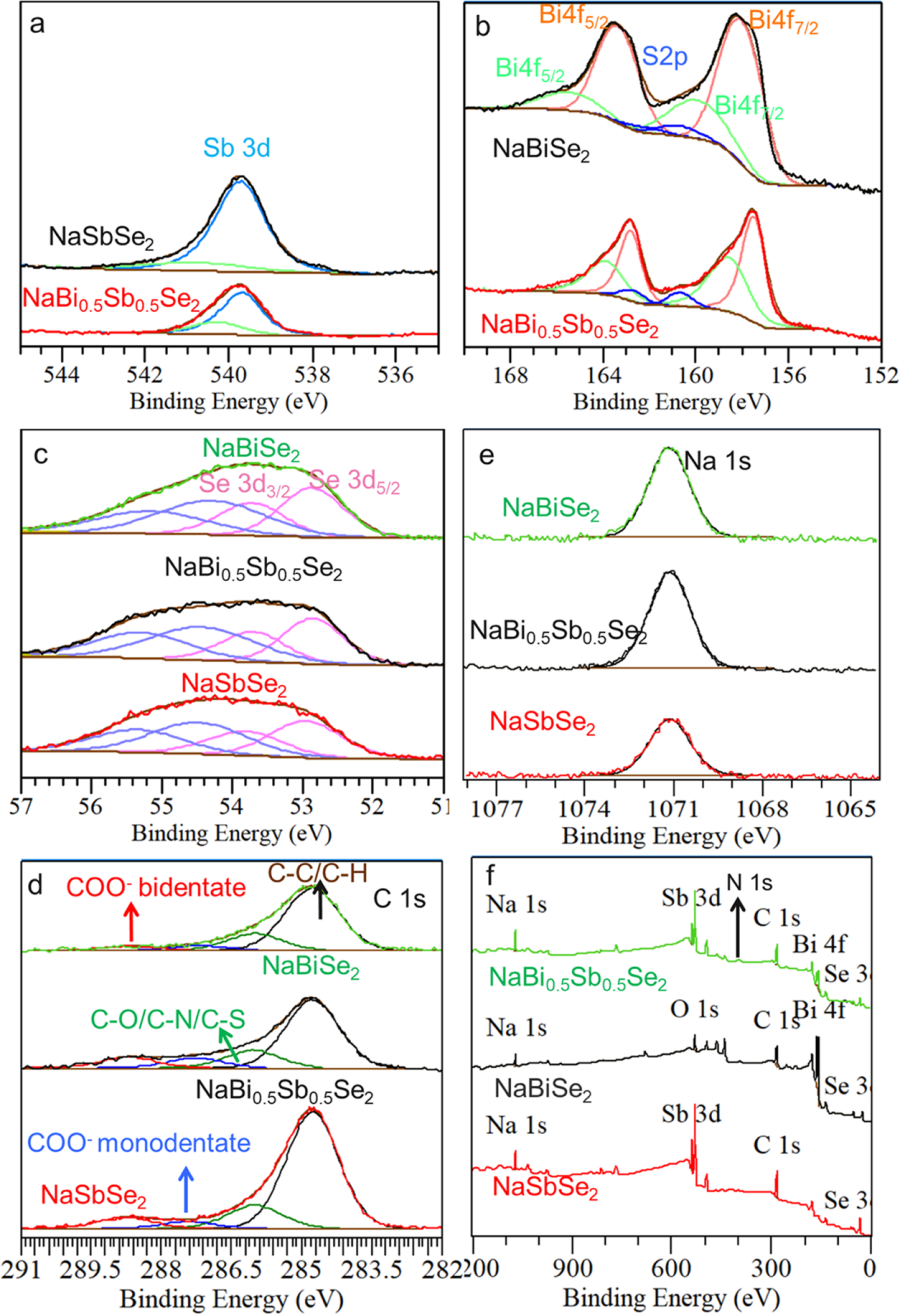
(a) XPS of Sb 3d for NaSbSe_2_ (black) and NaBi_0.5_Sb_0.5_Se_2_ (red), (b) XPS of Bi 4f for NaBiSe_2_ (black) and NaBi_0.5_Sb_0.5_Se_2_ (red), (c) XPS of Se 3d, (d) Na 1s, (f) C1s, and (e) survey for NaBiSe_2_ (green), NaBi_0.5_Sb_0.5_Se_2_ (black), and NaSbSe_2_ (red).

Further, from the XPS analysis of NaBiSe_2_, NaBi_0.5_Sb_0.5_Se_2_, and NaSbSe_2_ NCs, the elemental composition and surface chemistry of the NCs are corroborated (Figure 6). The low energy peaks of Sb (Sb 3d at ∼540 eV, Figure 6a), Bi (4f_5/2_ at ∼163 eV and Bi 4f_7/2_ at ∼158 eV, Figure 6b), and Se (Se 3d_5/2_ at ∼53 eV and Se 3d_3/2_ at∼54 eV, Figure 6c) and the peak of Na 1s at ∼1071 eV (Figure 6 d) correspond to the crystal bound Sb, Se, Bi, and Na for the respective NCs.^36,46^ Besides, Sb, Bi, and Se all show higher energy peaks that could be associated with interaction with surface-bound ligands.^47,48^ The high energy peaks of Sb 3d (green in 6a) and Bi 4f (green in 6b) possibly arise from the interaction of the Sb and Bi with the COO^–^ group of the surface-bound oleates. Similarly, the high energy peaks in the Se 3d XPS (54.5 and 55.3 eV) can be ascribed to the surface-bound alkane thiol–selenium complex (R-CH_2_S–Se_*n*–1_-Se^–^) formed upon the thiol–amine reaction with Se as observed in the IR spectra. In the C 1s (Figure 6e) XPS spectra, the higher energy peak at ∼286 eV compared to the C–C/C–H peak at ∼285 eV can be ascribed to C–O/C–N/C–S of the surface-bound oleate, oleylamine, and alkane thiol–selenium complex.^49^ The 287.3 and 288.7 eV peaks may arise from the C–OO^–^ of the surface-bound oleate showing monodentate and bidentate binding. Furthermore, the XPS survey of the NCs supports the presence of N from oleylamine as seen from N 1s XPS peak at ∼400 eV (Figure 6f).

**Figure 7:**
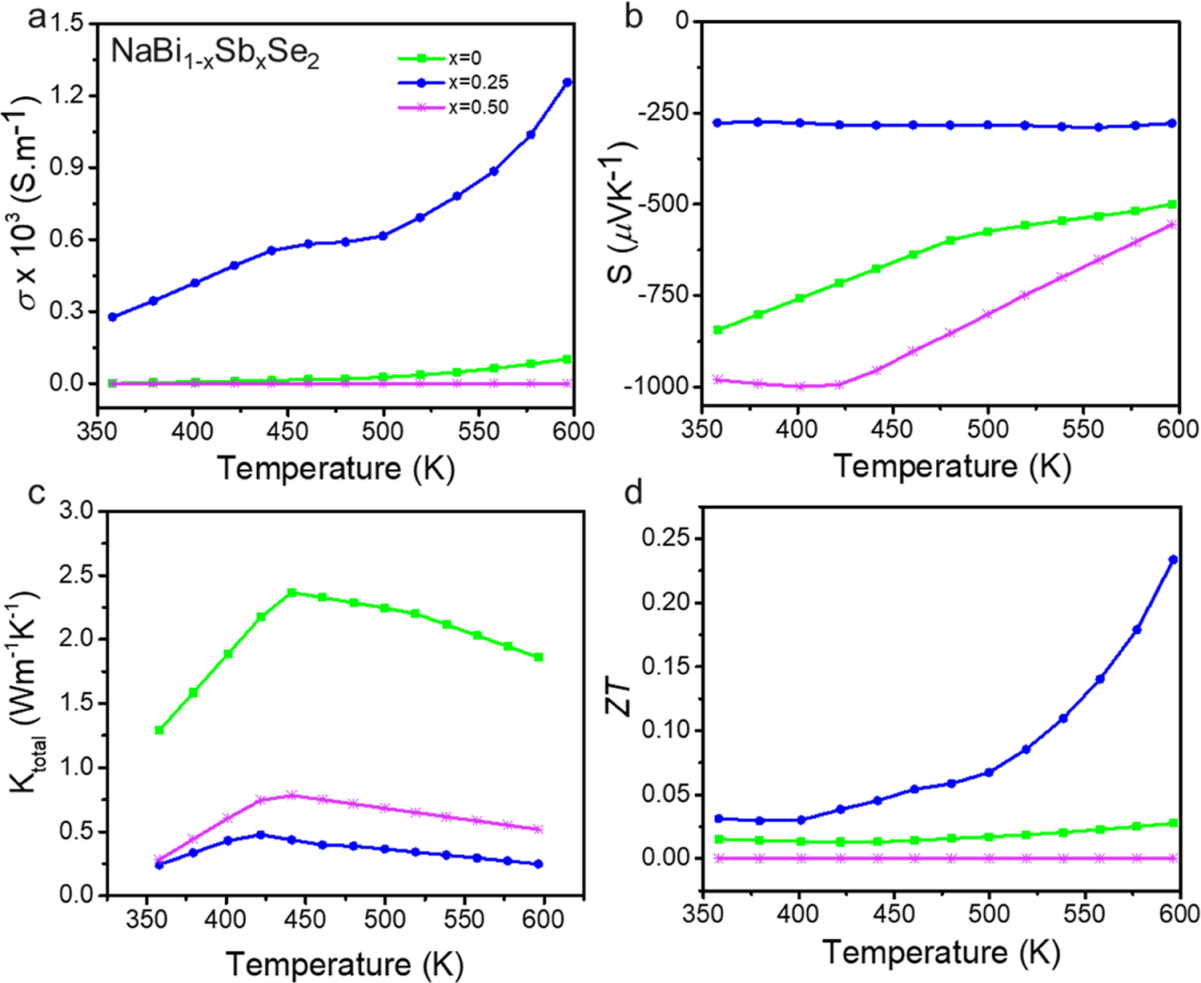
(a) Electrical conductivity, σ, (b) Seebeck coefficient, *S*, (c) thermal conductivity, *k*, and (d) TE figure of merit, *ZT*, of NaBi_1–*x*_Sb*_x_*Se_2_. *x* = 0, *x* = 0.25, and *x* = 0.5 are denoted by green, blue, and pink, respectively.

A highly disordered multinary composition increases the point defects to scatter phonons thus reducing the thermal conductivity of the material. Additionally, in NaBiCh_2_ (Ch = Se, S), the conduction band is dominated by the Bi p orbital, hence the free electron concentration can be modulated by Sb substitution. Thus, the TE properties of nanostructured NaBi_1–*x*_Sb*_x_*Se_2_ pellets obtained from the hot pressing of the NCs were investigated. For TE applications, a low thermal conductivity (*K*) and a high power factor *S*^2^σ are necessary to increase the thermoelectric figure of merit (*ZT*= *S*^2^σ*T*/*K*, where *S* = Seebeck coefficient and σ = electrical conductivity). However, the interdependent nature of these parameters makes the optimization harder. In NaBi_1–*x*_Sb*_x_*Se_2_, increasing the Sb substitution to *x* = 0.25, the carrier concentration increases as seen from the one order of magnitude rise in the electrical conductivity (σ) from 0.1 S·m^–1^ for *x* = 0 to 1.3 S·m^-1^ for *x* = 0.25 (Figure 7a). The negative Seebeck coefficient (*S*) values indicate that the electron is the majority charge carrier for these materials (*x* = 0, 0.25, and 0.5). The unsubstituted NaBiSe_2_ is characterized by high *S*, ranging from −844 mV·K^–1^ at 358 K to −499 mV·K^-1^ at 596 K. At a substitution of *x* = 0.25, the increase in the carrier concentration reduces the *S* as shown in Figure 7b. However, a further increase in Sb substitution (x=0.5) becomes detrimental for electrical conductivity and increases the Seebeck coefficient. Recently, the 5S^2^ LP in Sb was shown to be stereochemically more active than 6S^2^ LP in Bi.^36^ The increased LP activity upon Sb substitution induces local structural distortion to disrupt phonon propagation resulting in lower thermal conductivities (*K*). With *x* = 0.25 and 0.5, the thermal conductivity decreased below ∼0.8 W·m^–1^·K^–1^. Notably, with *x* = 0.25, a very thermal conductivity of 0.25 W·m^–1^·K^–1^ is achieved at ∼596 K (Figure 7c). The cumulative effect of the increased σ and the decreased *S* ensure a relatively high PF of 0.1 mW·m^–1^·K^–2^ at 596 K (Figure S13) which together with the low thermal conductivity resulted in promising *ZT* values of ∼0.24 at 596 K, well above the *ZT* values obtained from the unsubstituted material, at 0.03 (Figure 7d). Notably, no significant phase or compositional change occurred for the *x* = 0.25 sample (Figure S14). These results demonstrate this substitution approach is an effective strategy to tune the transport properties of multinary alkali metal–pnictogen chalcogenides.

## Conclusions

In summary, we have developed a systematic HI synthesis approach to produce NaBi_1–*x*_Sb*_x_*Se_2–*y*_S*_y_* NCs with controlled composition. The high reactivity of the Se-alkahest precursor used transformed the initial Bi^0^ NCs to form NaBiSe_2–*y*_S*_y_* NCs via Bi^3+^ diffusion into the nascent ternary NCs. Faster conversion of Bi^0^ NCs allowed a relatively narrow size distribution. By simply varying the Sb to Bi precursor concentration and S to Se concentration, the composition and shape of the multinary NaBi_1–*x*_Sb*_x_*Se_2_ and NaBiSe_2–*y*_S*_y_* NCs could be varied. Sb-rich NCs display spherical shapes, and S incorporation in NCs exhibits axial elongation along the <111> direction. The incorporation of Sb also increases the size of NCs. Furthermore, when the TE properties of the Sb-substituted n-type materials are studied, a significant reduction in thermal conductivity (below 0.8 W·m^–1^·K^–1^) is achieved compared to unsubstituted materials. Furthermore, optimization of the power factor in NaBi_0.75_Sb_0.25_Se_2_ increases the TE figure of merit (*ZT*) an order of magnitude compared to NaBiSe_2–*y*_S*_y_*. We anticipate that our synthetic findings will help us to optimize the functional properties of emerging ABE_2_ (A = alkali metal, B = Metal^3+^, and E = chalcogen) NCs, opening a range of new possibilities not only in the field of thermoelectrics but also in several other fields of application of these versatile materials.

## Experimental Section

### Chemicals

Sodium carbonate (Na_2_CO_3_, 97%, Lot# BCBW0418), bismuth acetate [Bi(OAc)_3_, 99%, Lot# NKPL6983V], selenium powder (Se, 99.99%, Lot# MKBV1065V), 1-dodecanethiol (1-DDT, ≥ 98%, Lot# STBF43147V), oleylamine (OLA, 70%, Lot# STBJ0354), 1-octadecence (ODE, 90%, Lot# NKBL4740V), oleic acid were purchased from Sigma-Aldrich. Toluene (Tol), methanol, and ethylacetate were purchased from Lennox, Ireland. The chemicals were used as received without any further purification. For making 0.2 M sodium oleate (NaOL), 255 mg (2.4 mmol) of Na_2_CO_3_ was mixed with 20 mL of ODE and 3 mL of OLA in a 3-neck round-bottom flask (RBF) and evacuated at 120 °C for 1h before heating it to 150 °C and annealing it for 3h.

The 0.5M Se-stock solution was prepared by stirring 10 mmol of Se in 10 mL of OLA and 10 mL of 1-DDT overnight in an Ar-filled glovebox.

### NaBiSe_2_ Nanocrystal (NC) Synthesis

In a typical synthesis, 77 mg (0.2 mmol) Bi(OAc)_3_ and 2 mL of NaOL were mixed with a solvent mixture of 1 mL of OLA and 4 mL of ODE in a RBF, and the reaction mixture was evacuated at 105 °C for 1h (5 min ramp to 105 °C and 1h soak). The vacuum pressure was kept below 200 mTorr during evacuation. Afterward, the reaction mixture was heated to 200 °C under an argon atmosphere (5 min ramp to 200 °C). 3 mL of Se-stock solution was injected when the temperature reached 200 °C. After the Se injection, the temperature drops to below 190 °C. When the temperature of the reaction vessel recovers to 200 °C, it was allowed to proceed for another 30 min of growth time. Afterward, the heating mantle was removed to terminate the reaction by natural cooling till 90 °C. Upon reaching 90 °C, 10 mL of toluene was mixed with the gel-like reaction mixture by sonication and vortexing well.

### NaBi_1–*x*_Sb*_x_*Se_2_ and NaBiSe_2–*y*_S*_y_* Nanocrystal (NC) Synthesis

In a typical synthesis, Bi(OAc)_3_ and Sb(OAc)_3_ with a molar ratio decided from desired stoichiometry (e.g., 0.15 mmol of Sb(OAc)_3_ and 0.05mmol of Bi(OAc)_3_ for achieving a composition of NaBi_0.25_Sb_0.75_Se_2_) and 2 mL of NaOL were mixed with a solvent mixture of 1 mL of OLA and 4 mL of ODE in a RBF, and the reaction mixture was evacuated at 105 °C for 1h (5 min ramp to 105 °C and 1h soak). The vacuum pressure was kept below 200 mTorr during evacuation. Afterward, the reaction mixture was heated to 240 °C under an argon atmosphere (5 min ramp to 240 °C). 3 mL of Se-stock solution was injected when the temperature reached 200 °C. When the temperature of the reaction vessel reached 240 °C, it was allowed to proceed for another 36 min of growth time for Bi/Sb=0.75/0.25, 40 min Bi/Sb= 0.5/0.5, 42 min for Bi/Sb=0.25/0.75, and 45 min for NaSbSe_2_. Afterward, the heating mantle was removed to terminate the reaction by natural cooling till 90 °C. Upon reaching 90 °C, 10 mL of toluene was mixed with the gel-like reaction mixture by sonication and vortexing well. For S substitution, the S-to-Se mole ratio was used based on stoichiometry. For example, to synthesize NaBiSe_1_S_1_, the chalcogen stock solution was prepared by stirring 5 mmol of Se and 5 mmol of S in 10 mL of OLA and 10 mL of 1-DDT, and 3 mL of the stock solution was injected at 200 °C with a growth temperature of 240 °C for 40 min. For NaBiSe_1.8_S_0.2_ and NaBiSe_1.4_S_0.6_, a growth time of 35 and 38 min was used, respectively.

### NC Purification Procedure

The NCs synthesized and mixed with 10 mL of toluene were poured into a 50 mL centrifuge tube and vortexed well. After that, the NC solution was mixed with IPA and sonicated for 10 min. The dispersed NCs in an equal amount of toluene and IPA were centrifuged at 5000 rpm for 5 min. The pellet was collected and dispersed in 10 mL of Tol first and 10 mL of IPA was further added, sonicated, and vortexed to disperse the NCs well. The NC solution was again centrifuged at 5000 rpm for 5 min, and the process was repeated another 2 times and dried at 80 °C overnight in vacuum.

For thermoelectric pellet fabrication, after the third wash, the nanocrystals were further mixed with 200 μL of butyl amine in the nanocrystal dispersed in 10 mL of toluene and mixed for 15 min via sonication. Afterward, 10 mL of IPA was added to the dispersion and further sonicated for 5 min and mixed via vortex for 1 min. The NC solution was centrifuged at 5000 rpm for 5 min, and the process was repeated 1 more time before drying overnight in a vacuum oven at 80 °C.

### General Safety and Handling

Safety considerations of each chemical should be thoroughly noted from safety data sheets (SDS are available on the chemical supplier webpage) before handling them. The ability to regulate vacuum and Ar-filled inert atmosphere in the Schlenk line is essential. Therefore, before performing any experiments, one should be well-equipped and experienced in air-free synthesis in high boiling point solvents at elevated temperatures and Schlenk line handling. All of the chemicals must be handled with proper personal protective equipment (PPE), especially lab coats, gloves, and safety goggles. All of the chemical substances should be handled/measured inside the glovebox or fume hood as per SDS. Among all of the chemicals, oleylamine is highly corrosive and toxic. Hence, it should be handled in the fume hood with proper PPE. Antimony acetate can be responsible for oral toxicity. Thus, it should be handled in a closed environment with appropriate PPE. 1-Dodecanethiol is corrosive and a skin irritant with a strong odor. Therefore, it should always be handled inside a fume hood. Any spillage should be cleaned immediately. The evacuation steps should be performed with a liquid N_2_ trap connection to condense hazardous gas evolved during the reaction. The Ar flow should be maintained using an outlet reservoir such as a bubbler. During the reaction, this will also help prevent direct exposure to evolved gaseous impurities and products. The hot sodium oleate transfer should be done cautiously with a glass syringe.

### Aliquot Study

During NC growth, 1 mL of solution from the RBF was withdrawn at desired temperature and time after Se injection. To ensure minimal depletion in precursor concentration, a maximum of 2 mL of reaction solution in total was withdrawn from the RBF. After withdrawal, the growth was immediately quenched by ejecting into 2 mL of Tol. The NCs in 2 mL of Tol were dispersed in 2 mL of IPA and centrifuged for 5 min at 5000 rpm, followed by another two cycles of redispersion in 2 mL of Tol and 2 mL of IPA and centrifugation at 5000 rpm for 3 min. For NMR characterization, the aliquot collected at 200 °C was thermally quenched and no toluene was added. After cooling it down to room temperature, the liquid portion of the aliquot was characterized through ^1^H NMR (JEOL 400 MHz NMR spectrometer) in CDCl_3_. The peaks were referenced to the residual chloroform peak at 7.26 ppm for ^1^H NMR.

### Electron Microscopy

For transmission electron microscopy (TEM) analysis, the NCs were dispersed in Tol and drop cast on continuous carbon-coated 200 mesh nickel grids. Low-resolution and high-resolution TEM (HRTEM) and dark-field scanning transmission electron microscopy (DFSTEM) were conducted by using a 200 kV JEOL JEM-2100F field-emission microscope, equipped with a Gatan UltraScan CCD camera and EDAX Genesis energy dispersive X-ray spectroscopy (EDS) detector. For analyzing the HRTEM data, interplanar distances and particle orientation were determined from the selected area FFT analysis using GMS3 software.

### X-ray Diffraction (XRD) Analysis

XRD of drop-cast films of the NCs on the flat surface of p-type boron-doped silicon zero background was conducted using a PANalytical Empyrean instrument equipped with a Cu Kα radiation source (λ = 1.5418 Å) and a 1-D X’celerator strip detector with a diffractometer operating at 40 kV and 40 mA. All of the PXRD patterns of the final NCs were analyzed by the Rietveld method in HighScore Plus software using a pseudo-Voigt profile.

### X-ray photoelectron spectroscopy (XPS)

X-ray photoelectron spectroscopy (XPS) was carried out using a Kratos AXIS ULTRA spectrometer fitted with a mono Al Kα (1486.58 eV) X-ray gun. Calibration was performed using the C 1s line at 284.8 eV, while construction and peak fitting were performed using CasaXPS software. XPS was performed on the vacuum-dried nanostructure samples.

### Thermoelectric Measurements

The Seebeck coefficient and resistivity were simultaneously measured under a helium atmosphere in an LSR-3 Linseis system. All samples were tested for at least three heating and cooling cycles. Considering the system and measurement accuracy and measurement accuracy, we estimated the measurement error of conductivity and Seebeck coefficient to be about 4%. Thermal conductivities were obtained by multiplying the thermal diffusivity (λ), the constant pressure heat capacity (*C*_p_), and the density of the material (ρ): *K*_total_ = λC_p_ρ. Thermal diffusivities were measured by a Xenon Flash Apparatus XFA 600 and a Laser Flash Analyzer LFA 1000, Linseis, which have an estimated error of ca. 5%. The heat capacity was estimated from the Dulong–Petit limit (3R law). The densities were calculated from Archimedes’ method which is ∼90% of the theoretical density.

## Supporting Information Available

Additional data of SEM, TEM, HRTEM, STEM-EDS mapping, ^1^H NMR, XRD patterns, and Rietveld refinement of the aliquot, and nanocrystals samples and additional data of thermal transport property measurement (PDF)
