# Supplementary material for: Colloidal Synthesis of Multinary Alkali-Metal Chalcogenides Containing Bi and Sb: An Emerging Class of I–V–VI2 Nanocrystals with Tunable Composition and Interesting Properties
Source: Chem Mater. 2023 Jun 8;35(12):4810–20. doi: 10.1021/acs.chemmater.3c00673 (PMC10308588; doi:10.1021/acs.chemmater.3c00673)
Supplement: Supplementary file 1 — cm3c00673_si_001.pdf [file cm3c00673_si_001.pdf]

Supporting information

Colloidal Synthesis of Multinary Alkali-metal Chalcogenides Containing Bi and Sb - an Emerging Class of I-V-VI_2 Nanocrystals with Tunable Composition and Interesting Properties

Nilotpal Kapuria [1], Bingfei Nan [2], Temilade Esther Adegoke[1], Ursel Bangert [3], Andreu Cabot [2], Shalini Singh[1], Kevin M Ryan[1]**

1 Department of Chemical Sciences and Bernal Institute, University of Limerick, V94T9PX Limerick, Ireland

2 Catalonia Institute for Energy Research -IREC, 08930 Barcelona, Spain; ICREA, 08010 Barcelona, Spain

3 Department of Physics and Energy and Bernal Institute, University of Limerick, V94T9PX Limerick, Ireland

Email: Kevin.M.Ryan@ul.ie, Shalini.Singh@ul.ie

Table of content:

1.1. XRD analysis of $NaBiSe_2$ NCs 3

Supporting information

1.2. ICP-OES analysis of the NCs **3**

1.3. XRD analysis of the S-substituted nanocrystals **4**

1.4. XRD analysis of the Sb-substituted nanocrystals **5**

1.5. ^{1}H NMR analysis of aliquot before Se addition **6**

1.6. TEM analysis of aliquot before Se addition **7-8**

1.7. XRD analysis of the aliquots of $NaBiSe_2$ NCs **8**

1.8. TEM analysis of aliquot of $NaBiSe_2$ system collected after Se addition **8-9**

1.9. TEM analysis of aliquot of $NaBi_{0.5}Sb_{0.5}Se_2$ system collected after Se addition **10**

1.10. XRD and TEM analysis of aliquot of $NaBi_{0.5}Sb_{0.5}Se_2$ system collected after Se addition **11**

1.11. Power factor of Sb substituted NCs **12**

1.12. Post characterization of the $NaBi_{0.75}Sb_{0.25}Se_{2-y}S_y$ material-based pellet **13**

1.13. XPS analysis of 200 °C aliquot before Se addition **14**

1.14. Thiol-amine-Se reaction **14**

Supporting information

1.1. XRD analysis of $NaBiSe_2$ NCs

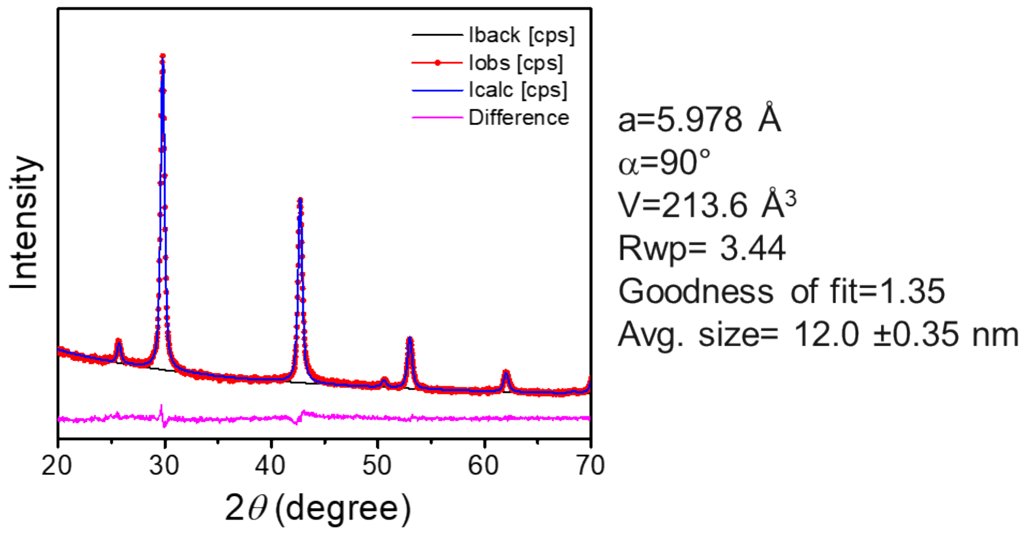

Figure S1.Rietveld refinement of $NaBiSe_2$ NCs.

1.2. Table S1. ICP-OES analysis of the NCs

Supporting information

Substituted element % in $NaBiSe_2$	Elemental composition by ICP-OES				
Elements	Na	Bi	Sb	Se	S
$NaBiSe_2$	24.0± 0.9%	27.2± 1.5%	--	45.7± 1.8 %	3.1 ± 0.3%
$NaBiSe_{1.8}S_{0.2}$	24.0 ± 0.7%	25.9 ± 0.8%	--	44.3 ± 1.6%	5.8 ± 0.4%
$NaBiSe_{1.4}S_{0.6}$	22.3 ± 0.8%	25.9 ± 0.9%	--	36.8 ± 1.0%	15.0 ± 0.5%
$NaBiSe_1S_1$	26.5 ± 0.8%	26.7± 1.0%	--	25.4 ± 0.4%	21.4 ± 0.5%
$NaBi_{0.75}Sb_{0.25}Se_2$	24.2 ± 0.2%	19.6 ± 1.7%	5.9 ± 0.4%	48.6 ± 2.8%	1.7 ± 0.3%
$NaBi_{0.5}Sb_{0.5}Se_2$	24.4 ± 1.6%	12.7 ± 1.2%	11.7 ± 0.9%	47.7 ± 3.2%	3.5 ± 0.2%
$NaBi_{0.25}Sb_{0.75}Se_2$	24.2 ± 1.9%	6.4 ± 0.9%	18.9 ± 2.1%	48.8 ± 5.0%	1.7 ± 0.2%
$NaSbSe_2$	24.0 ± 0.9%	--	27.2 ± 3.3%	45.7 ± 3.3%	3.1 ± 0.2%

1.3. XRD analysis of the S-substituted nanocrystals.

Supporting information

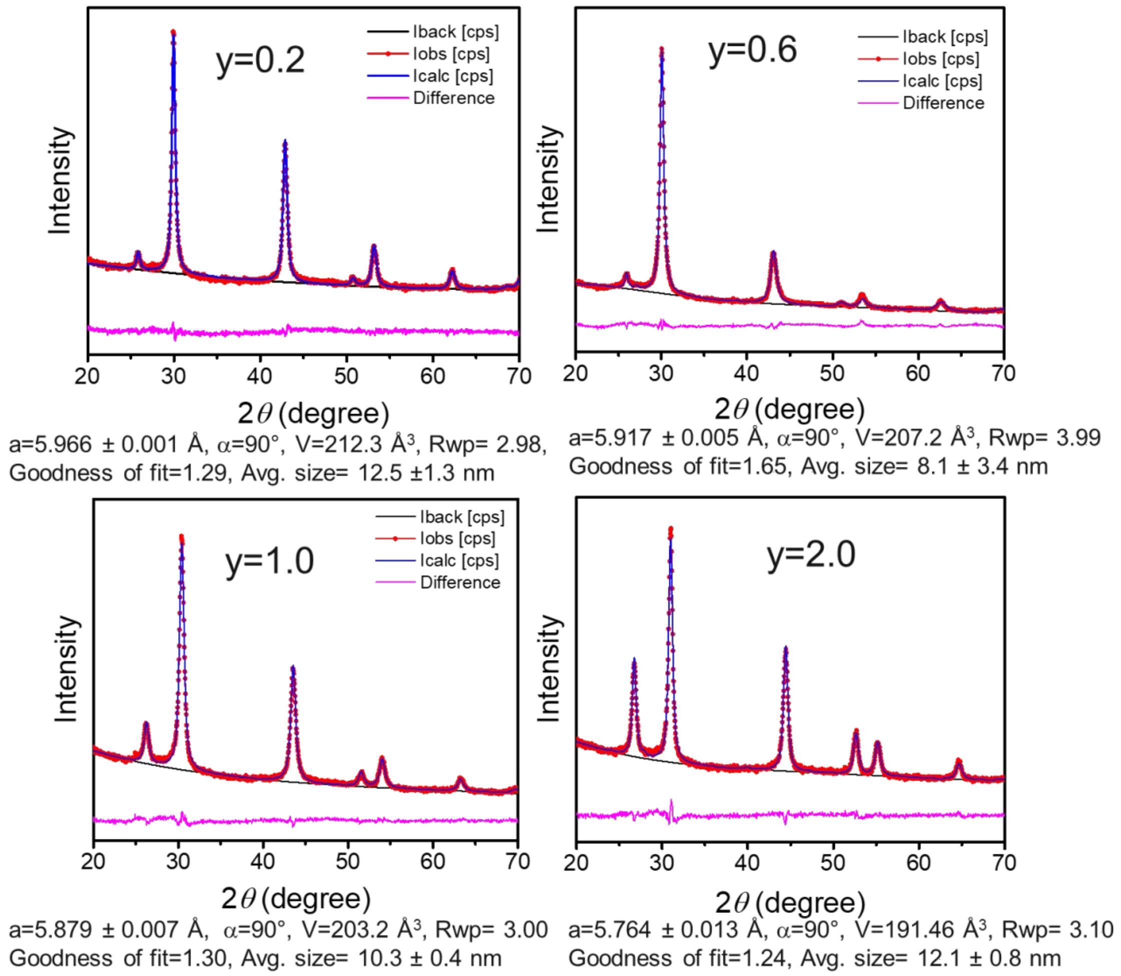

Figure S2. Rietveld refinement of $NaBiSe_{2-y}S_y$ NCs.

1.4. XRD analysis of the Sb-substituted nanocrystals

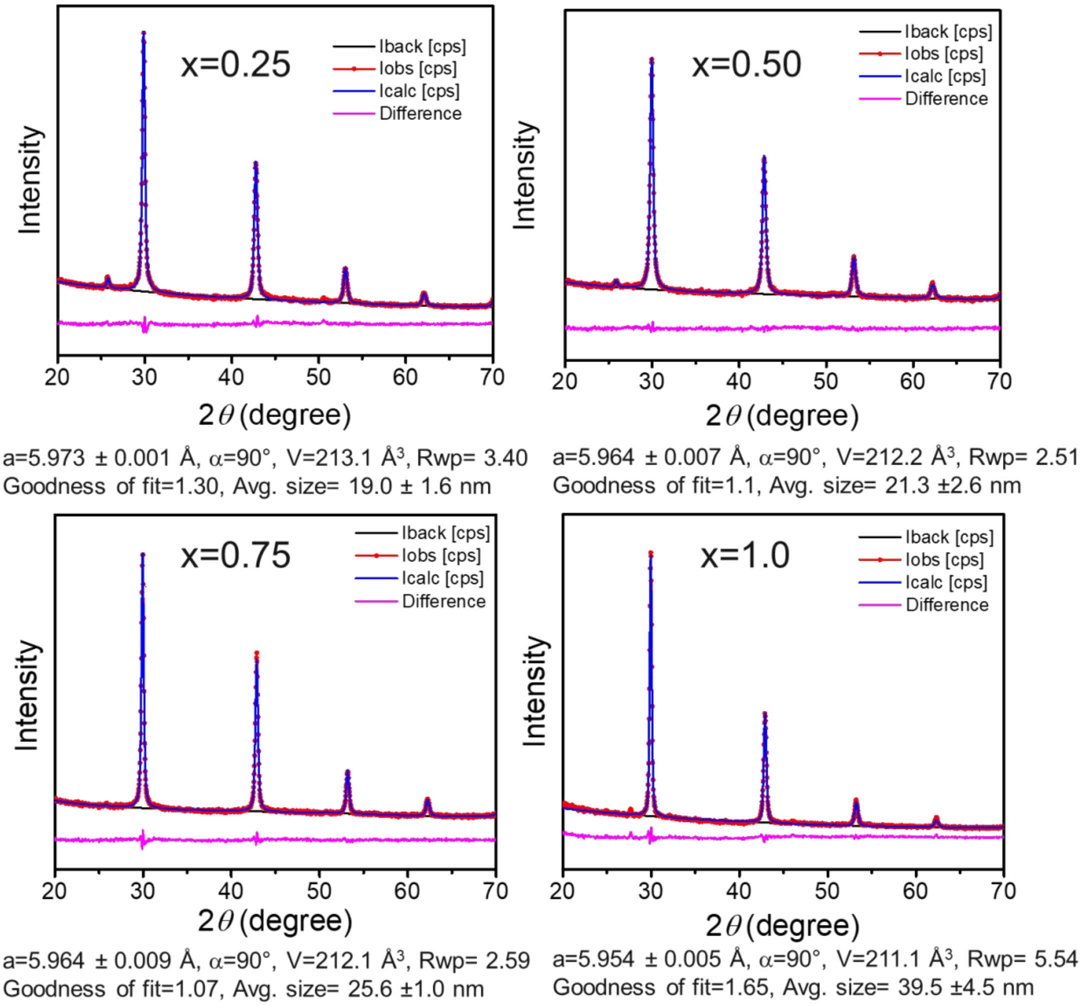

Figure 3. Rietveld refinement of $NaBi_{1-x}Sb_xSe_2$ NCs.

1.5. ^{1}H NMR analysis of aliquot before Se addition in $NaBiSe_2$ system

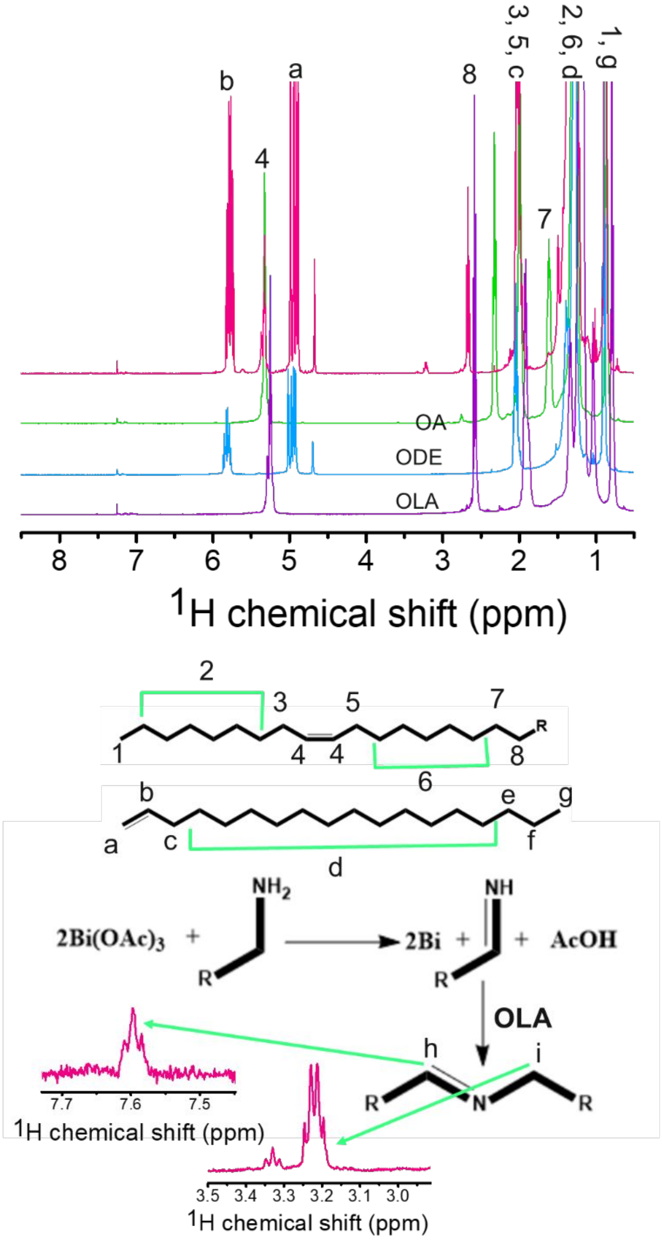

Figure S4. ^{1}H NMR of the aliquot supernatant withdrawn before Se introduction. $CDCl_3$ used as reference displaying peak at 7.26 ppm.

1.6. TEM analysis of aliquot before Se addition

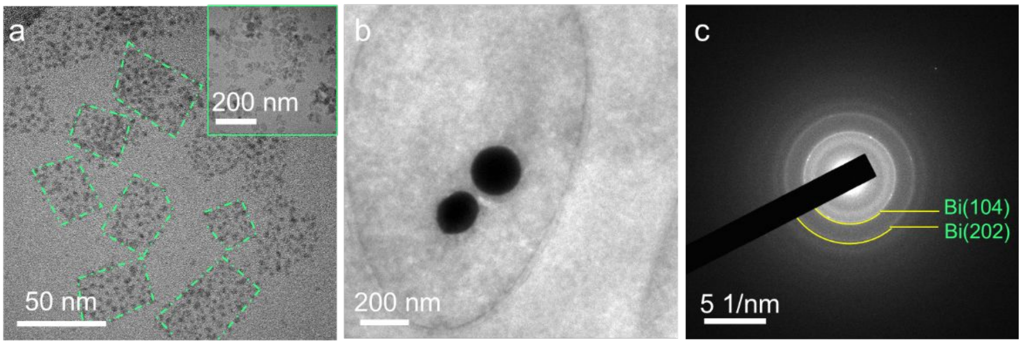

Figure S5. (a) TEM image of quasi-cubic nanophases, (b) nanophases with Bi NCs; (c) SAED pattern of nanophases with Bi showing Bi crystal peaks.

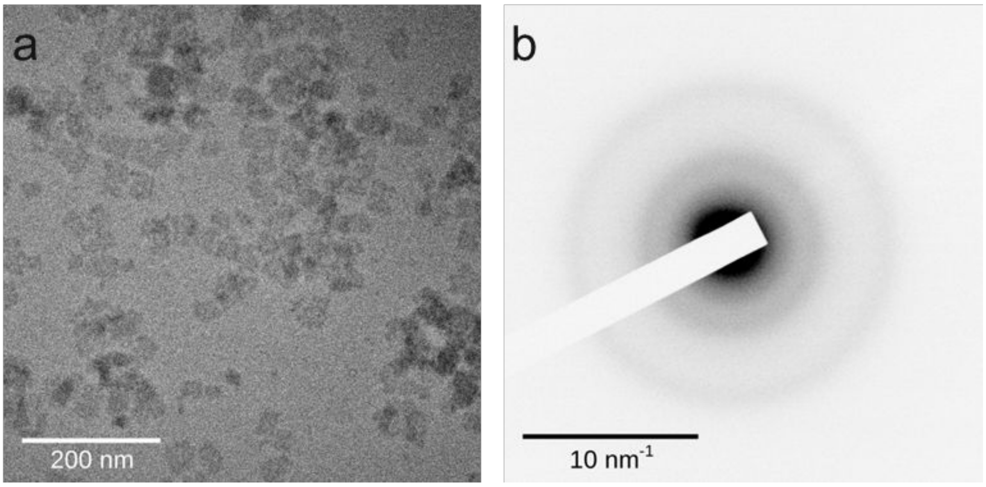

Figure S6. (a) TEM image of quasi-cubic nanophases, and corresponding (b) SAED pattern.

1.7. XRD analysis of the aliquots of $NaBiSe_2$ NCs

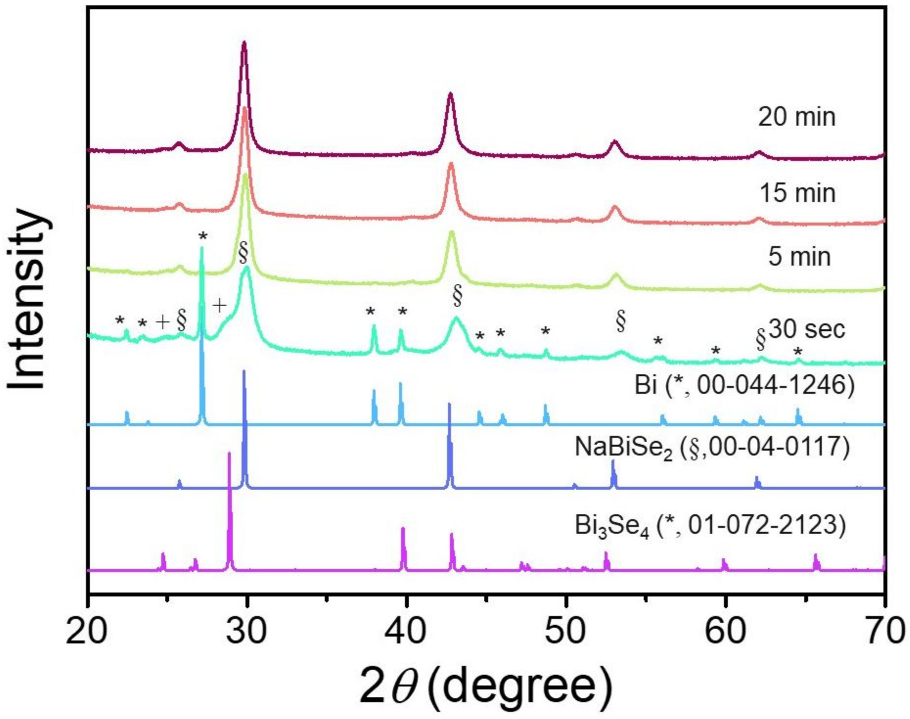

Figure S8. XRD pattern of the washed aliquots withdrawn different growth times.

1.8. TEM analysis of aliquot of $NaBiSe_2$ system collected after Se addition

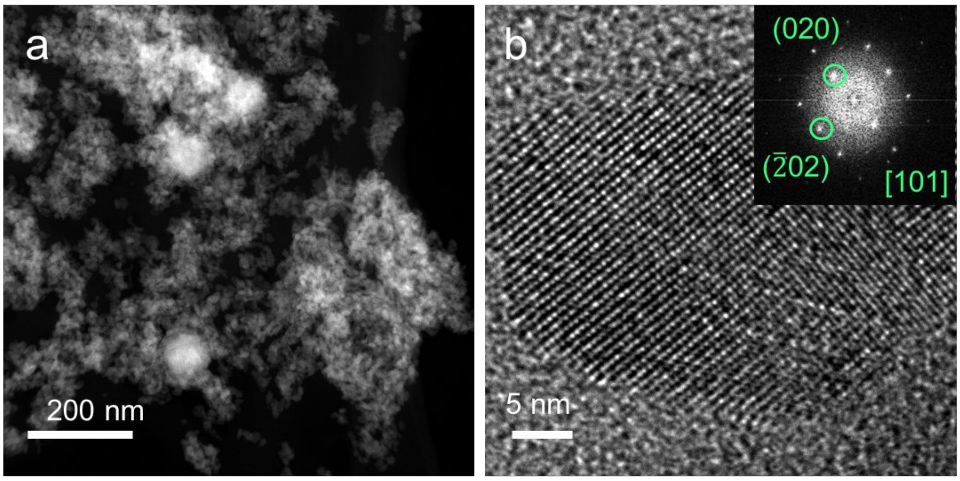

Figure S9. (a) ADF-STEM image of the aliquot withdrawn after 30 sec of Se introduction, (b) HRTEM of a $NaBiSe_2$ NC in the same aliquot.

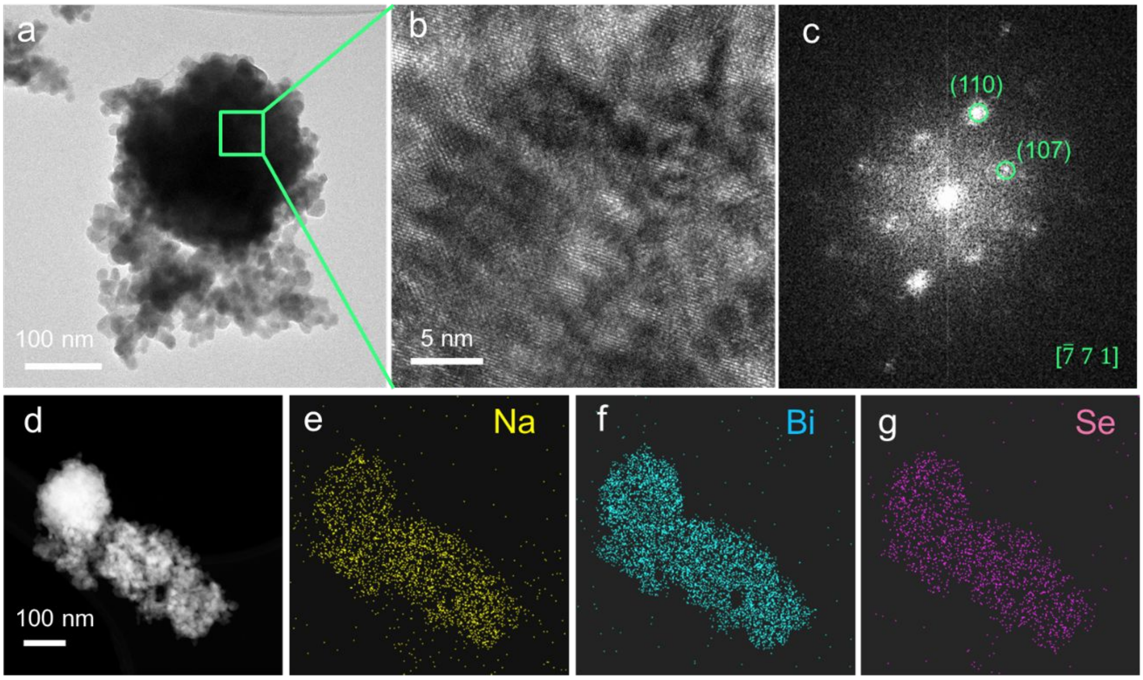

Figure S10. (a) Low magnification TEM image of transformed larger NPs in the aliquot collected after 30 sec of Se introduction, (b) HRTEM image of a segment of the NPs and corresponding (c) FFT pattern confirming the phase similarity with rhombohedral Bi_3Se_4 (d) STEM-EDS elemental maps of the secondary NCs showing maps for (e)Na, (f)Bi, (g)Se.

1.9. TEM analysis of aliquot of $NaBi_{0.5}Sb_{0.5}Se_2$ system collected before Se addition

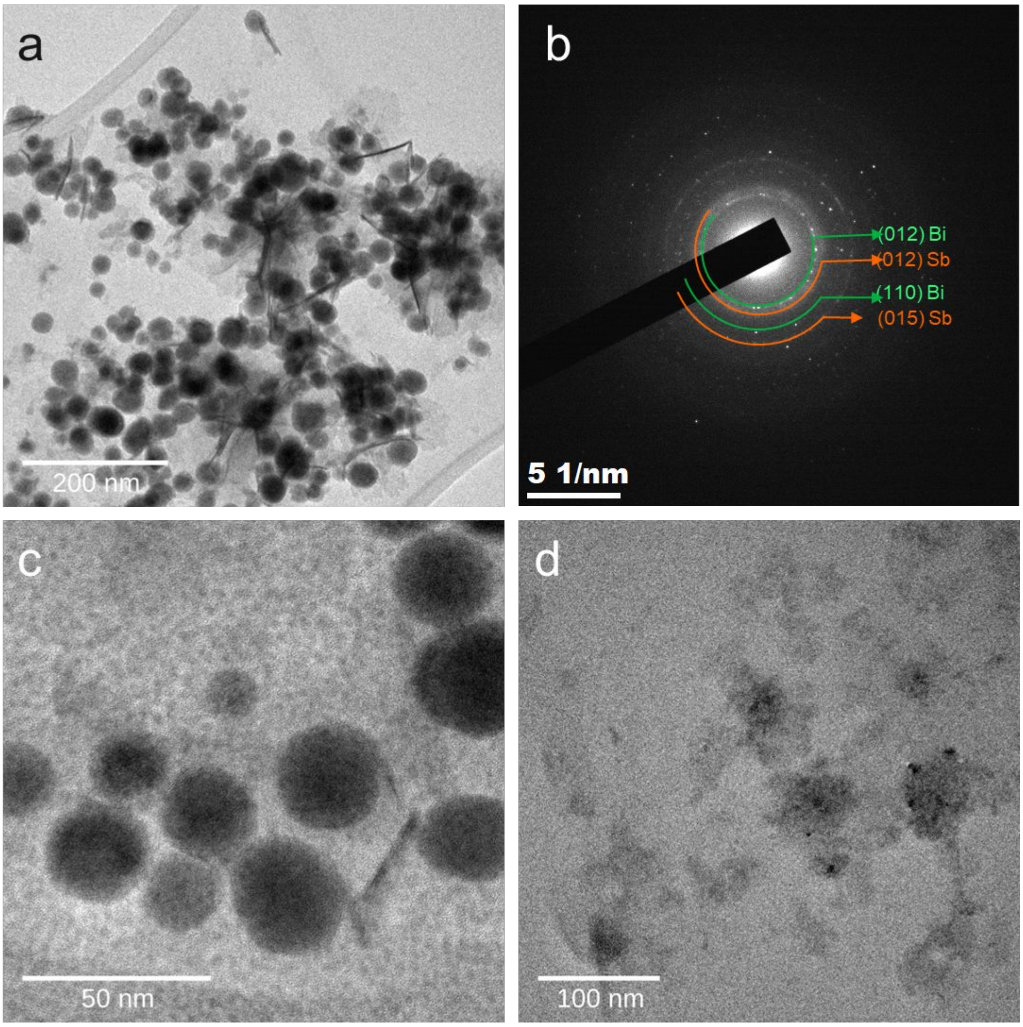

Figure S11. (a) TEM image of the aliquot collected at 200 ˚C before Se addition from $NaBi_{0.5}Sb_{0.5}Se_2$ system, (b) their corresponding SAED pattern showing the presence of Bi and Sb NCs. (c) Amorphous NPs with Sb and Bi NCs; (d) Amorphous NPs.

1.10. XRD and TEM analysis of aliquot of $NaBi_{0.5}Sb_{0.5}Se_2$ system collected after Se addition

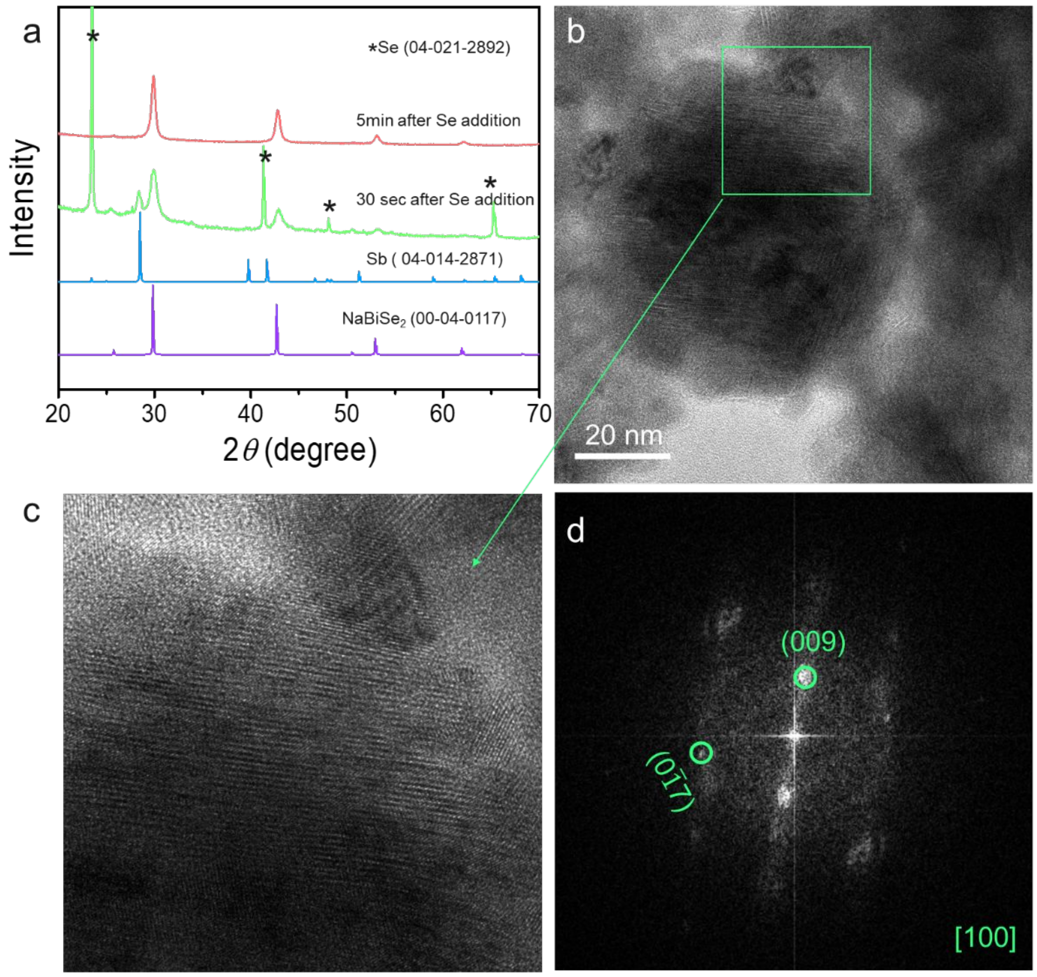

Figure S12. (a) XRD pattern of the aliquot collected after Se addition from $NaBi_{0.5}Sb_{0.5}Se_2$ system, (b) TEM characterization of Bi_3Se_4 NC, (c) HRTEM of a selected area from the NC and the corresponding (d) FFT pattern.

1.11. Power factor of Sb substituted NCs.

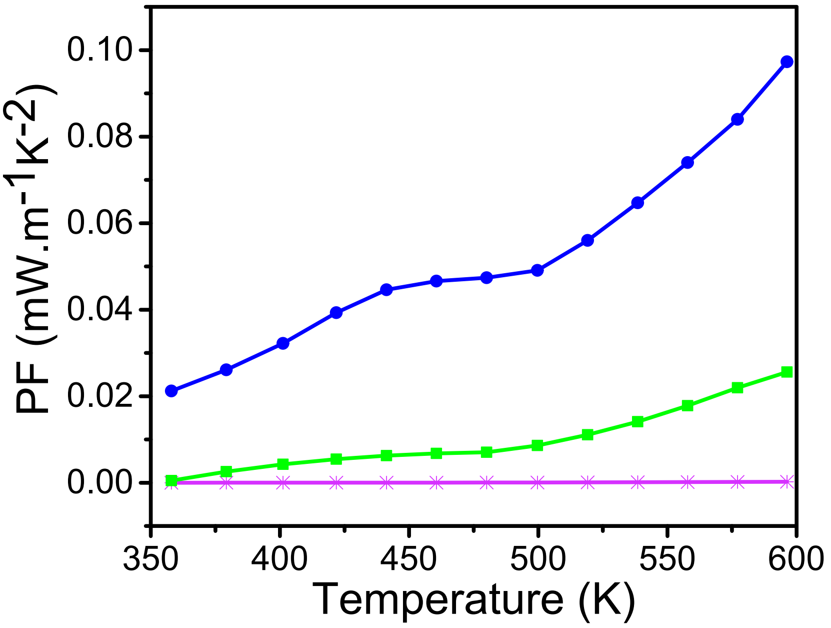

Figure S13. Power factor of $NaBi_{1-x}Sb_xSe_2$ NCs. x=0, x=0.25, x=0.5 are denoted by green, blue and pink respectively.

Supporting information

1.12. Post characterization of the $NaBi_{0.75}Sb_{0.25}Se_{2-y}S_y$ material-based pellet

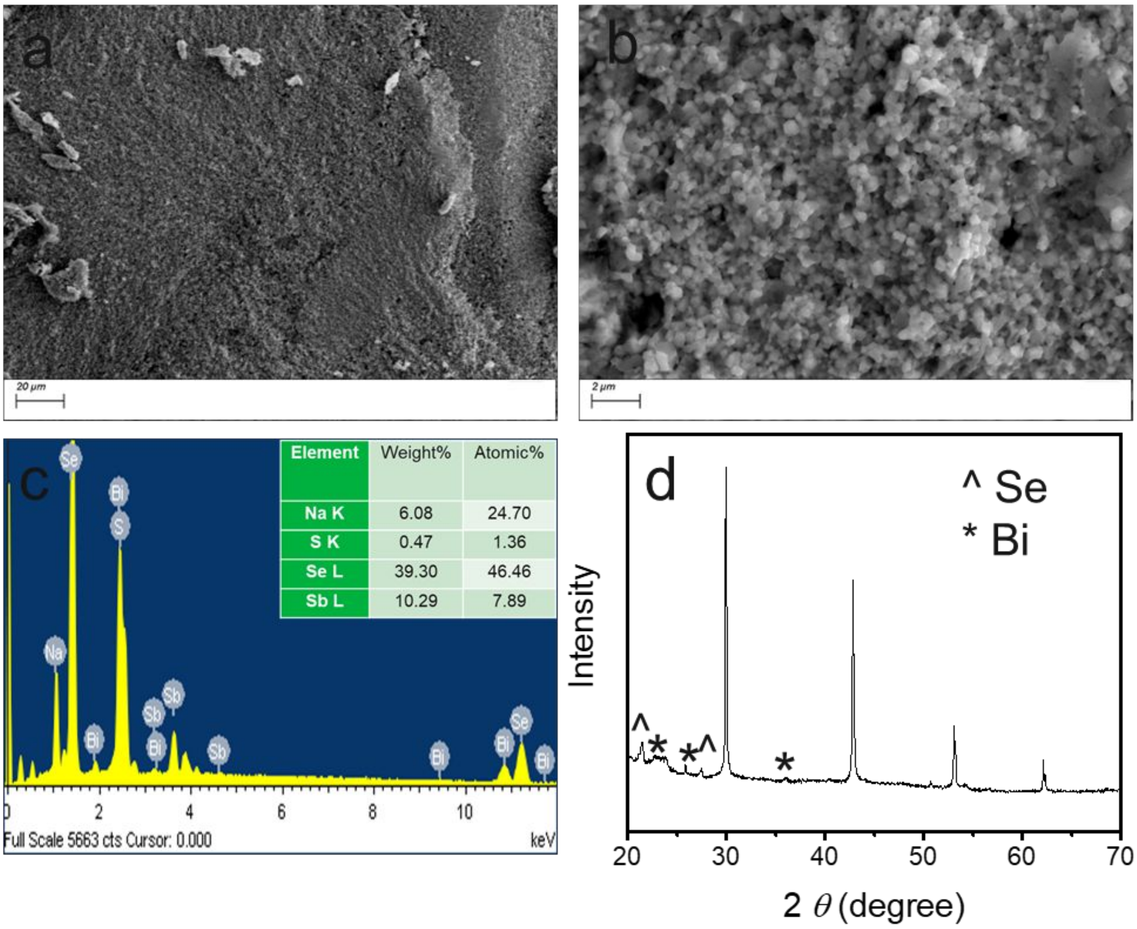

Figure S14. (a, b) SEM image and (c)SEM-EDS spectra and (d) XRD pattern of $NaBi_{0.75}Sb_{0.25}Se_{2-y}S_y$ material based pellet.

1.13. XPS analysis of 200 ˚C aliquot before Se addition.

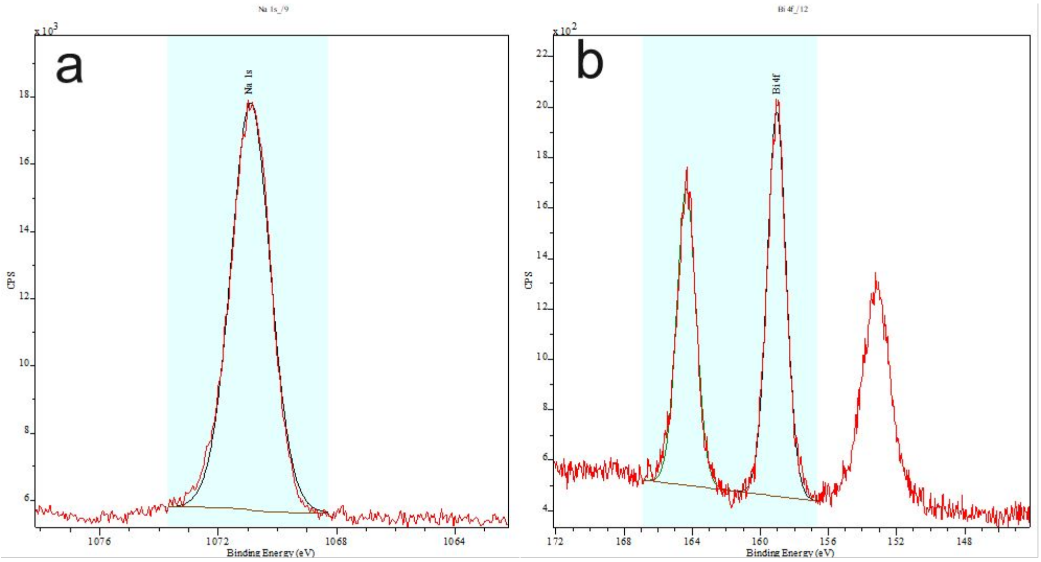

Figure S15. XPS analysis of the drop cast thin film (on glass substrate) of the 200 ˚C aliquot withdrawn before Se addition. (a) The peak at 1071 eV corresponds to Na 1s of Na-based amorphous NPs, (b)the peaks at ~ 159 and 164 eV correspond to Bi 4f of metallic bismuth.

1.14. Thiol-amine-Se reaction

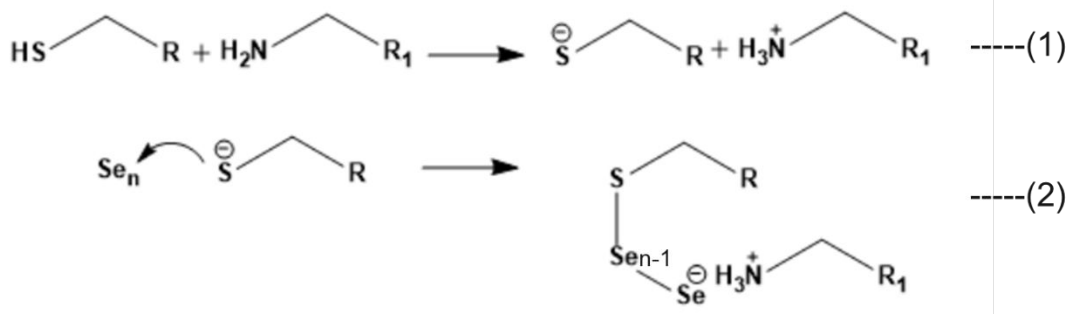

Figure S16. Reaction mechanism of alkane thiol-selenium complex formation.
